# AIM2 inflammasome activation may mediate high mobility group box 1 release in murine allergic rhinitis

**DOI:** 10.1016/j.bjorl.2020.12.014

**Published:** 2021-02-15

**Authors:** Yan Wang, Shan Chen, Ping-Li Yang, Jian-Jun Chen, Wei-Jia Kong, Yan-Jun Wang

**Affiliations:** a Huazhong University of Science and Technology, Tongji Medical College, Union Hospital, Department of Otorhinolaryngology, Wuhan, China; b Shihezi University School of Medicine, The First Affiliated Hospital, Department of Otorhinolaryngology, Shihezi, China; c Huazhong University of Science and Technology, Tongji Medical College, Union Hospital, Institute of Otorhinolaryngology, Wuhan, China

**Keywords:** AIM2, Nlrp3, Caspase-1, Inflammasome, Allergic rhinitis, Pyroptosis

## Abstract

**Introduction:**

High mobility group box 1 protein participates in the pathogenesis of allergic rhinitis. Activation of the inflammasome can mediate the release of high mobility group box 1. The role of the absent in melanoma 2 inflammasome in allergic rhinitis remains unclear.

**Objective:**

This study aimed to investigate the function of absent in melanoma 2 inflammasome in murine allergic rhinitis and the interaction between high mobility group box 1 and the absent in melanoma 2 inflammasome.

**Methods:**

A murine allergic rhinitis model was established using twenty Balb/c mice. Expression of the components of the absent in melanoma 2 inflammasome: absent in melanoma 2, apoptosis-associated speck-like protein containing a CARD (Asc), caspase-1 p20, and additional nod-like receptor family pyrin domain containing 3 (Nlrp3) were detected by western blotting during allergic rhinitis. Alterations of absent in melanoma 2, caspase-1, and high mobility group box 1 after ovalbumin challenge were demonstrated by immunohistochemistry. TdT-mediated dUTP Nick end labeling, TUNEL assay, and cleavage of caspase-3 and PARP-1 were used for the observation of pyroptosis.

**Results:**

Eosinophilia and goblet cell infiltration were observed in the nasal mucosa of mice in the allergic rhinitis group. Absent in melanoma 2, Asc, and caspase-1 p20 increased after ovalbumin exposure while Nlrp3 did not. High mobility group box 1 was released in the nasal mucosa of allergic rhinitis mice. TUNEL-positive cells increased in the epithelium and laminae propria, whereas cleavage of caspase-3 and PARP-1 was not observed.

**Conclusions:**

The absent in melanoma 2 inflammasome was activated and pyroptosis may occur in the nasal mucosa after ovalbumin treatment. These may contribute to the translocation of high mobility group box 1 and the development of allergic rhinitis.

## Introduction

Allergic rhinitis (AR) is defined as a type of nasal mucosa inflammation associated with an IgE-mediated immune response against allergens.^1^ Patients with AR are usually characterized by symptoms of nasal obstruction, rhinorrhea, sneezing and nasal itching.^2^ The disorder also causes a considerable burden to patients, affecting their quality of life, cognitive function, and self-perception.^3^ Nowadays, AR has become an important worldwide public health problem affecting 10%–20% of the adult population.^4^ Understanding the nature of AR will improve the care of patients suffering from this disorder.

It is widely accepted that high mobility group box 1 (HMGB1), a ubiquitously-expressed protein, plays multiple roles in physiological and pathological processes.^5^ HMGB1 was originally described as an important chromatin protein,^6^ but is now known to have different functions based on compartment-specific expression.^7^ When HMGB1 is released into an extracellular location, it mediates activation of the innate immune response as a pro-inflammatory factor.^8^ Our previous study demonstrated that HMGB1 increased in the nasal mucosa and trans-located after ovalbumin (OVA) exposure in a murine AR model. Inhibition of HMGB1 by ethyl pyruvate exerted a therapeutic effect on AR.^9^ However, the mechanism of HMGB1 release in AR remains unclear.

HMGB1 can be passively released from dying cells,^10^ as well as being actively secreted by inflammatory cells such as macrophages, monocytes, dendritic cells, and natural killer cells.^11^ Numerous pathways have been demonstrated to be implicated in the release of HMGB1, such as double-stranded RNA-activated protein kinase (PKR)-mediated inflammasome activation,^12^ and chromosome region maintenance 1-mediated nuclear translocation.^13^ Recently, a series of studies revealed that caspase-1 could act as a potential therapeutic target in AR using various protective agents,^14^, ^15^, ^16^ suggesting that the inflammasome may be activated after allergen exposure. Activation of the inflammasome leads to pyroptosis and results in cell death,^17^ which then meditates HMGB1 release.^18^ However, there is a lack of research into which the subfamily of inflammasome is activated in AR. Assembly of an inflammasome complex is initiated by nucleotide-binding domain, leucine-rich repeat receptor (NLR) or absent in melanoma 2 (AIM2) after recognizing pathogen-associated molecular patterns (PAMPs) or danger-associated molecular patterns (DAMPs).^19^ Activated NLRs or AIM2 can recruit apoptosis-associated speck-like protein containing a caspase-recruitment domain (ASC) to engage caspase-1 activation.^20^ Nlrp3 and AIM2 have been demonstrated to be up-regulated in the pathogenesis of upper airway disease such as acute and chronic rhinosinusitis.^21^, ^22^

We hypothesized that the inflammasome is activated in the nasal mucosa of mice by exposure to OVA, and that pyroptosis may occur after inflammasome activation. To test this hypothesis, we established a mouse AR model and examined activation of the inflammasome in AR.

## Methods

### Animals

Twenty wild-type male BALB/c mice aged from 6 to 8 weeks were involved in the study. The animals were housed in a specific pathogen-free animal facility. These mice were randomly divided into two groups – control group and AR group – with 10 mice in each group. Six mice from each group were used for analysis of protein expression, and the remaining mice were used for histological observation and immunohistochemistry. The experiments were conducted according to the National Institutes of Health Guidelines for the Care and Use of Laboratory Animals and were approved by the Institutional Animal Care and Use Committee (IACUC Number: S2372).

### Establishment of a murine allergic rhinitis model

**Figure 1:**
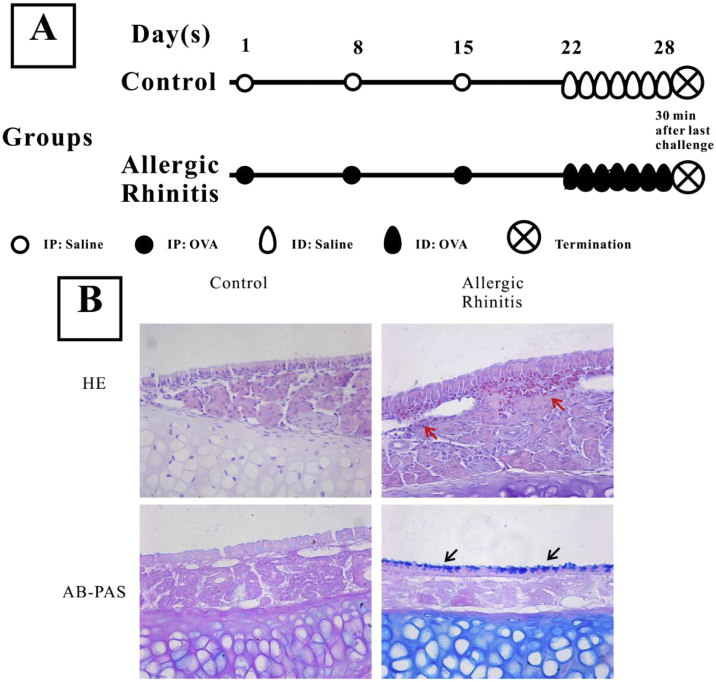
Establishment of a murine model of Allergic Rhinitis (AR). Mice in the AR group were sensitized by intraperitoneal (IP) injection of 40 μg OVA and 2 μg aluminum on experimental days 1, 8, and 15, and were challenged with OVA by daily inhalation (ID) from days 22 to 28 (A). H&E staining and AB-PAS staining were performed to observe the eosinophilic infiltration and goblet cell hyperplasia. Evident infiltration of eosinophils (red arrows) and increased numbers of goblet cells (black arrows) were observed in the AR group (B) compared with the control group.

The establishment of mouse AR was performed as previously described.^9^ The experimental group was injected intraperitoneally on the first, 8th, and 15thdays of the study with 200 μL PBS solution containing 40 μg OVA (grade V; Sigma-Aldrich; St. Louis, MO, USA) and 2 mL aluminum hydroxide, while the control group was only given the same amount of PBS solution. From day 22, 500 μg OVA in 20 μL PBS was inhaled into the bilateral nasal cavities through normal breathing daily for one week (Fig. 1) in the AR group, while the control group received saline alone.

### Histological observation and immunohistochemistry

Four mice from each group were used for analysis of activation of the inflammasome and HMGB1 localization. The mice were humanely killed after anesthesia and immediately decapitated. The snouts were fixed with 4% formaldehyde overnight, decalcified with 10% ethylenediaminetetraacetic acid-2 Na in PBS for one month, dehydrated in a graded ethanol series and then embedded in paraffin. Finally, the samples were sectioned at 5 um and collected.

For the analysis of protein expression, immunohistochemical analysis was performed with a REAL™ Envision™ Detection Kit (Dako, Glostrup, Denmark). All procedures were performed according to the manufacturer’s instructions. After deparaffinization and dehydration, the tissue sections underwent heat-induced epitope retrieval. The tissues were incubated with 4% H_2_O_2_ to block endogenous peroxidases after being washed three times with PBS. Bovine serum albumin was used to saturate any excess protein-binding sites. The blocked sections were then incubated with antibodies against AIM2 (diluted 1:200), caspase-1 (diluted 1:50), or HMGB1 (diluted 1:250), at 4 °C overnight. The next day, a secondary antibody was applied to the sections for 1 h at room temperature, and the slides were again washed three times with PBS after incubation. Finally, the slides were counterstained with hematoxylin, and then observed under a light microscope. To observe eosinophilic infiltration and goblet cell hyperplasia, sections were stained with Hematoxylin & Eosin (H&E) and Alcian Blue & Periodic Acid Schiff stain (AB-PAS) as previously described.^9^

### TdT-mediated dUTP nick end labeling (TUNEL)

TUNEL assay was performed based on the manufacturer’s instructions. After deparaffinization and dehydration, sections were washed three times with PBS, then incubated with proteinase K dissolved in Tris−HCl solution (20 μg/mL, pH 7.4) for 30 min at 37 °C. After washing another three times with PBS, a 50 μL volume of the TUNEL reaction mixture consisting of 48 μL of label solution and 2 μL of TdT solution was added to the slides and incubated for 90 min at 37 °C, and the sections were again washed three times with PBS. Finally, the sections were counterstained with DAPI and observed under a fluorescence microscope (Leica Microsystems Ltd., Wetzlar, Germany).

### Western blot analysis

The protein expressions of HMGB1 and components of inflammasomes were examined by western blotting. Total protein was extracted from the nasal mucosa samples using RIPA lysis buffer and protein concentrations were measured by BCA assay. Protein lysates were separated on 12% Sodium Dodecyl Sulfate (SDS) – Polyacrylamide gels and transferred to Polyvinylidene Difluoride (PVDF) membranes. The membranes were blocked for 1 h with 5% fat-free milk in Tris-Buffered Saline containing Tween (TBST) and incubated overnight at 4 °C with the appropriate dilution of primary antibodies: anti-Nlrp3 (R&D Systems, Minneapolis, MN, USA, diluted 1:300), anti-AIM2 (Abcam, Cambridge, MA, USA, diluted 1:1,000), anti-Asc (Abcam, diluted 1:1,000), anti-caspase-1 (Biovision, Milpitas, CA, USA, 1:200), and anti-β-actin (Millipore, Billerica, MA, USA, diluted 1:3,000). After washing, the membranes were incubated for 1 h at room temperature with the appropriate HRP-conjugated secondary antibody (diluted 1:3,000). The protein bands were visualized by BeyoECL Plus (Beyotime, Haimen, Jiangsu, China), and Gel-Pro analyzer 4.0 software (Media Cybernetics, Inc., Rockville, MD, USA) was used for relative quantification, with β-actin as the internal control.

### Statistical analysis

All results are expressed as mean ± SD. Statistical analysis was performed using the statistical package for the social sciences (SPSS Version 18.0; SPSS Inc., Chicago, IL, USA). Student’s *t*-test was used to analyze the expression of inflammasome components. A value of *p* < 0.05 was considered statistically significant.

## Results

### Establishment of a murine AR model

H&E staining showed that numerous eosinophils infiltrated the nasal mucosa of AR mice, while no evident inflammatory cells were observed in that of control mice (Fig. 1). Furthermore, goblet cell hyperplasia was also observed in AR mice.

### Activation of the AIM2 inflammasome

**Figure 2:**
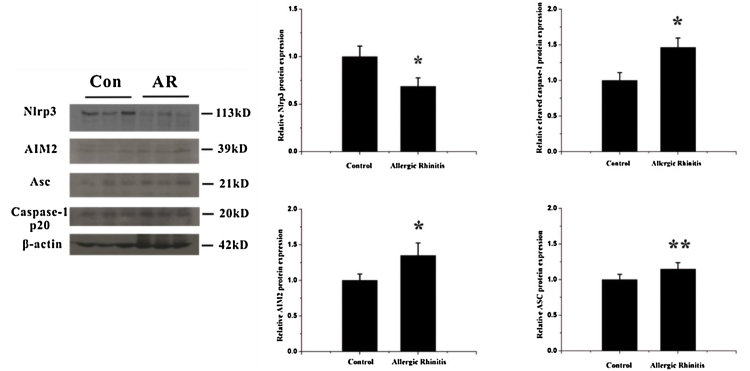
Expression of components of the inflammasome after OVA stimulation. The expressions of two receptors, Nlrp3 and AIM2, were analyzed by western blotting. The expression of Nlrp3 decreased whereas AIM2 increased in the nasal mucosa after OVA exposure, *p* <  0.01. Meanwhile the expression of another component of the inflammasome complex, Asc, and the levels of cleaved caspase-1 were both increased, *p* <  0.05 and 0.01, respectively (*p <  0.01, **p <  0.05).

**Figure 3:**
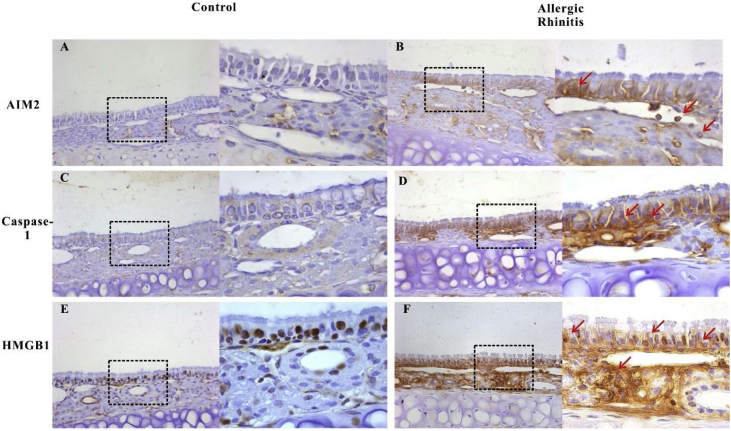
Expression of AIM2, caspase-1, and HMGB1 in the nasal mucosa of mice. AIM2 and caspase-1 were rarely expressed in the nasal mucosa from the control group (A, C). However, after OVA stimulation, the expression of both proteins was up regulated in the epithelium, lamina propria, and infiltrated inflammatory cells (B, D). HMGB1 was abundantly expressed in the epithelium and lamina propria, and mainly located in the nucleus in mucosa from control mice (E). The expression of HMGB1 significantly increased in the nasal mucosa of AR mice and the translocation of HMGB1 from the nucleus to the cytoplasm was observed (F, red arrows).

To detect alterations in expression of inflammasome components in the OVA-induced airway response, we observed the localization and expression of inflammasome components using immunohistochemistry and western blotting. The expressions of AIM2, asc, and cleaved caspase-1 were all significantly increased after OVA exposure (Figure 2, Figure 3).

### Pyroptosis but not apoptosis may occur after OVA stimulation

**Figure 4:**
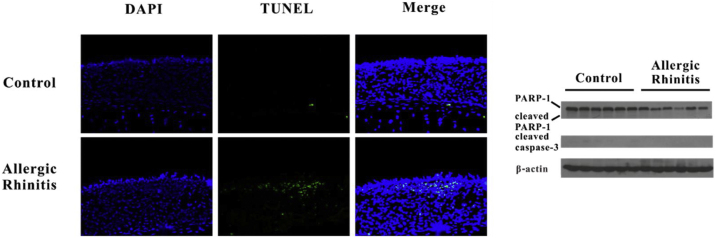
Pyroptosis but not apoptosis may occur in murine Allergic Rhinitis (AR). TUNEL assay revealed that fragmentation of DNA significantly increased in the nasal mucosa of mice in the AR group. However, there was no evident cleavage of caspase-3, and no PARP-1 was found in the AR group.

HMGB1 may be passively released as a result of cell pyroptosis. We detected DNA fragments using TUNEL and confirmed cleavage of caspase-3 and PARP-1. Increased numbers of TUNEL-positive cells were found in the AR group, whereas no differences in cleavage of caspase-3 or PARP-1 were observed between the two groups. The translocation of HMGB1 was observed in the AR group as a result of inflammasome activation (Fig. 4).

## Discussion

We successfully established an OVA-induced murine model of AR, which is characterized by Th2-dominated airway inflammation. Eosinophilia and goblet cell hyperplasia were found in the nasal mucosa of AR mice. In addition, significantly increased activation of AIM2 inflammasomes was found in the AR mice, where cytoplasmic transfer and up-regulation of HMGB1 were also observed.

In this study, we demonstrate activation of the AIM2 inflammasome in the nasal mucosa in AR. AIM2 has been found to act as a DNA sensor in innate immunity. Upon sensing cytosolic DNA, including bacterial, viral^23^ and host,^24^ the AIM2 protein recruits the adaptor protein ASC through the PYRIN-PAAD-DAPIN (PYD) domain and the ASC protein recruits procaspase-1 through the caspase activation and recruitment domain (CARD) to form an active AIM2 inflammasome. Our results showed that the components of the AIM2 inflammasome including AIM2, asc, and cleaved caspase-1 were up-regulated after OVA exposure in the nasal mucosa, suggesting that the AIM2 inflammasome was activated in murine AR. Recently, an interesting study carried out on the eosinophil extracellular traps showed that extracellular DNA concentrations in bronchoalveolar lavage of OVA-treated mice were significantly increased compared with controls.^25^ This result suggested that eosinophils may contribute to activation of the AIM2 inflammasome in AR. Furthermore, we analyzed cell death using the TUNEL assay in nasal mucosa and demonstrated an increase in cell death in the nasal sections of OVA- versus saline-treated animals. We also observed cleavage of caspase-3 and PARP-1, which are characteristic of apoptosis, in the nasal mucosa after OVA exposure. However, there is no evidence that cleavage of caspase-3 and PARP-1 occurred in the nasal mucosa after OVA stimulation. During pyroptosis, cells undergo chromosomal DNA cleavage and nuclear condensation and become positive in the TUNEL assay,^26^ but without the cleavage of typical caspase substrates such as caspase-3 and PARP that are characteristic of apoptosis.^27^ Therefore, we concluded that pyroptosis occurred in the nasal mucosa of our murine AR model. HMGB1 release can occur downstream of inflammasome formation and caspase-1 activation. Consistent with our previous finding, HMGB1 was released into the cytosol and extracellular space after OVA exposure. Released HMGB1 may induce and amplify AR. Thus, activation of the AIM2 inflammasome may induce HMGB1 translocation in AR.

Nlrp3 is the most studied structural subset of the inflammasome and plays an important role in the pathogenesis of numerous airway diseases such as asthma and acute lung injury.^28^ In our study, we found that Nlrp3 was downregulated in nasal mucosa from the AR group compared to the control group. An interesting study comparing the expression of NLRs in nasal biopsies from healthy individuals and patients suffering from intermittent AR showed that Nlrp3 was significantly lower in the allergic group, especially during pollen season, than among the healthy group.^29^ In another study using a murine asthma model, decreased expression of Nlrp3 was also found during airway inflammation.^30^ These results indicated that Nlrp3 may be down-regulated after allergen exposure as a defense mechanism. Furthermore, the effects of Th2 cytokines such as IL-4 and IL-13 on the expression of Nlrp3 were studied on human keratinocytes, THP-1 cells and mouse bone marrow-derived macrophages.^31^, ^32^ The research demonstrated that expression of Nlrp3 was suppressed by treatment with Th2 cytokines. Th2-dominated airway inflammation is one characteristic of AR, and Th2 cytokines such as IL-4, -5, and -13 are released after allergen stimulation.^33^ Therefore, the expression of Nlrp3 may be suppressed by Th2 cytokines in AR.

In our study, we demonstrated that the AIM2 inflammasome was activated in AR and may contribute to the release of HMGB1. During airway inflammation, numerous cell types participated in the development of AR, such as Th2 cells. Recently, a study on the role of Nlrp3 in Th2 differentiation revealed that it is expressed by CD4+ T-cells and acts as a key transcription factor.^34^ Thus Nlrp3 may be implicated in the pathogenesis of Th2-dominated diseases such as AR and asthma. However, a major limitation of our study is that the mucosa is a complex organ comprised of numerous tissues. It is difficult to show changes in protein expression of Nlrp3 at the level of a single cell type in the nasal mucosa. The role of Nlrp3 in AR thus warrants further study.

## Conclusion

In conclusion, we demonstrated that the AIM2 inflammasome was activated and pyroptosis may occur in the nasal mucosa after OVA treatment. The release of HMGB1 was also observed. Activation of the inflammasome may contribute to the translocation of HMGB1 and the development of AR. Caspase-1 may therefore act as a potential therapeutic target for AR.

## Funding

This study was supported by the 10.13039/100007452Wu Jieping Medical Foundation (no. LC1345) and The 10.13039/501100003819Natural Science Foundation of Hubei Province, China (no. 2014CFB359).

## Conflicts of interest

The authors declare no conflicts of interest.
